# Excellence of the closed intensive care unit system in critically ill surgical patients

**DOI:** 10.1371/journal.pone.0285035

**Published:** 2023-04-26

**Authors:** Seung Min Baik, Na Rae Yang, Tae Yoon Kim, Kyung Sook Hong

**Affiliations:** 1 Division of Critical Care Medicine, Department of Surgery, Ewha Womans University Mokdong Hospital, Ewha Womans University College of Medicine, Seoul, Korea; 2 Department of Surgery, Korea University College of Medicine, Seoul, Korea; 3 Department of Neurosurgery, Ewha Womans University Mokdong Hospital, Ewha Womans University College of Medicine, Seoul, Korea; 4 Division of Critical Care Medicine, Department of Surgery, Ewha Womans University Seoul Hospital, Ewha Womans University College of Medicine, Seoul, Korea; University of Pittsburgh Medical Center Pinnacle Health Medical Services, UNITED STATES

## Abstract

**Background:**

Despite reports that the closed intensive care unit (ICU) system improves clinical outcomes, it has not been widely applied for various reasons. This study aimed to propose a better ICU system for critically ill patients by comparing the experience of open surgical ICU (OSICU) and closed surgical ICU (CSICU) systems in the same institution.

**Methods and findings:**

Our institution converted the ICU system from “open” to “closed” in February 2020, and enrolled patients were classified into the OSICU and CSICU groups at that time from March 2019 to February 2022. A total of 751 patients were categorized into the OSICU (n = 191) and CSICU (n = 560) groups. The mean age of the patients was 67 years in the OSICU group and 72 years in the CSICU group (*p* < 0.05). The acute physiology and chronic health evaluation II score was 21.8 ± 7.65 in the CSICU group, which was higher than the score 17.4 ± 7.97 in the OSICU group (*p* < 0.05). The sequential organ failure assessment scores were 2.0 ± 2.29 in the OSICU group and 4.1 ± 3.06 in the CSICU group (*p* < 0.05). After correction for bias by logistic regression analysis for all-cause mortality, the odds ratio in the CSICU group was 0.089 (95% confidence interval [CI]: 0.014–0.568, *p* < 0.05).

**Conclusions:**

Despite considering the various factors of increased patient severity, a CSICU system is more beneficial for critically ill patients. Therefore, we propose that the CSICU system be applied worldwide.

## Introduction

Although there are various definitions of a critically ill patient, it refers to a patient requiring intensive care due to life-threatening multiorgan dysfunction that can result in morbidity and mortality. Moreover, the intensive care unit (ICU) refers to a well-organized special space that allows these critically ill patients to receive appropriate treatment. The ICU is characterized by a concentration of medical personnel for patient treatment, monitoring equipment capable of continuous monitoring, and providing various modalities such as mechanical ventilation and continuous renal replacement therapy (CRRT) essential for patient treatment [1].

Although the treatment of critically ill patients has been practiced for a long time, the study of critical care medicine itself is a new field that has been discussed “independently” around the world less than 100 years ago [2, 3]. During the Second World War or polio outbreak, the need for various treatment equipment and systems, including ventilator care, emerged, and the number of ICUs increased rapidly [4]. Accordingly, a standardized education and certification system for critical care medicine was introduced, and after that, reports on treatment results by specialists residing in the ICU continued, suggesting an ideal ICU manpower composition and operation method.

In the case of the Republic of Korea, as the Korean Society of Emergency Medicine was reorganized into the Korean Society of Critical Care Medicine in 1996 [5], research on critical care medicine suited to the Korean situation continued. In 2009, the intensivist system was implemented through the approval of the relevant society, and in 2015, as a policy, it was recommended that the intensivist reside in the ICU of a tertiary general hospital.

The number of intensivists has steadily increased since the implementation of the first intensivist system in 2009, with the number of intensivists being 1774 in 2022 in Korea. However, when the intensivist system for critical care medicine was first implemented in 2009, only 24 out of 1040 physicians (2.3%) were surgeons, and there has been a fluctuating trend since then, although it is gradually increasing, with only approximately 7% of the total intensive care subspecialists being surgeons as of 2022 [6].

According to a study investigating the reasons why surgeons are reluctant to become intensivists for critically ill surgical patients, it was reported that this was because of a decrease in satisfaction due to mismatch with the specialty of “surgery” as surgeons had fewer surgical opportunities. Moreover, the pressure of work continuously exposed to critically ill patients and the conflicts that may arise in the process of managing critically ill patients were reasons for the reluctance to become intensivists [7].

The ICU system is typically categorized into open ICU and closed ICU according to the degree of treatment intervention of the intensivist, in addition to the following ICU systems [8]: intensivist comanagement, hybrid, or transitional ICU or semi-closed ICU model, multiple consultant model and mixed ICU models, and neurosciences ICU. In the open ICU system, patients are cared for under the management of existing primary care physicians such as surgeons and internists, and, if required, critical care is selectively implemented through consultation with an intensivist [9]: In the closed ICU system, the intensivist takes complete responsibility for the management of patients admitted to the ICU and makes all clinical decisions, including admission, and discharge.

Intensivists are advantageous for the management of critically ill patients due to their high understanding of the pathophysiology of critically ill patients, evidence-based management, use of systematic treatment protocols, and high proficiency in high-level treatments such as CRRT and mechanical ventilation. Therefore, there are several reports that the closed ICU system in which the intensivist completely manages critically ill patients improves the clinical outcome of these patients [10–15].

However, despite these positive reports, a truly closed ICU system, which is managed by the ICU staff composed of intensivists, has not been widely established around the world. This is because of the difficulty to apply it uniformly due to differences in the level of medical care, number of doctors, medical facilities, medical finance, and the insurance system around the world. In particular, in the case of the surgical ICU, there is another reason. The anthropologist Joan Cassell reported that in the case of surgeons, it is often difficult to transfer the responsibility for patients to other doctors because they have a relationship where they share a promise of treatment with patients and caregivers through a special procedure known as surgery [16, 17]. Moreover, recent studies report that conflict and confusion often arise when medical staff that lacks knowledge about surgery and postoperative recovery process and complications treats patients [18].

Regarding Korea, the closed surgical ICU (CSICU) system is currently not widely applied due to manpower problems and medical resource problems, although the primary patients admitted to the surgical ICU are surgical patients. Nevertheless, due to the severe shortage of surgeons and the large regional imbalance, it is anticipated that the positive impact of actively applying the CSICU system will be large [19].

However, only a few studies have examined the effect of applying the CSICU system. Moreover, most studies were performed in the integrated ICU, which did not differentiate between the medical ICU and surgical ICU. Nevertheless, it is important to investigate the effects of the CSICU system under detailed conditions such as medical and surgical classification. This is because of the large difference in medical resources in each country and even within a country; the implementation of medical policies such as the application of the ICU system must be decided in consideration of various detailed situations.

This study aimed to propose an ICU system that is more beneficial for critically ill surgical patients by comparing the clinical outcomes of institutions that have experience with both the open surgical ICU (OSICU) and CSICU systems.

## Methods

### Patients and data collection

**Fig 1:**
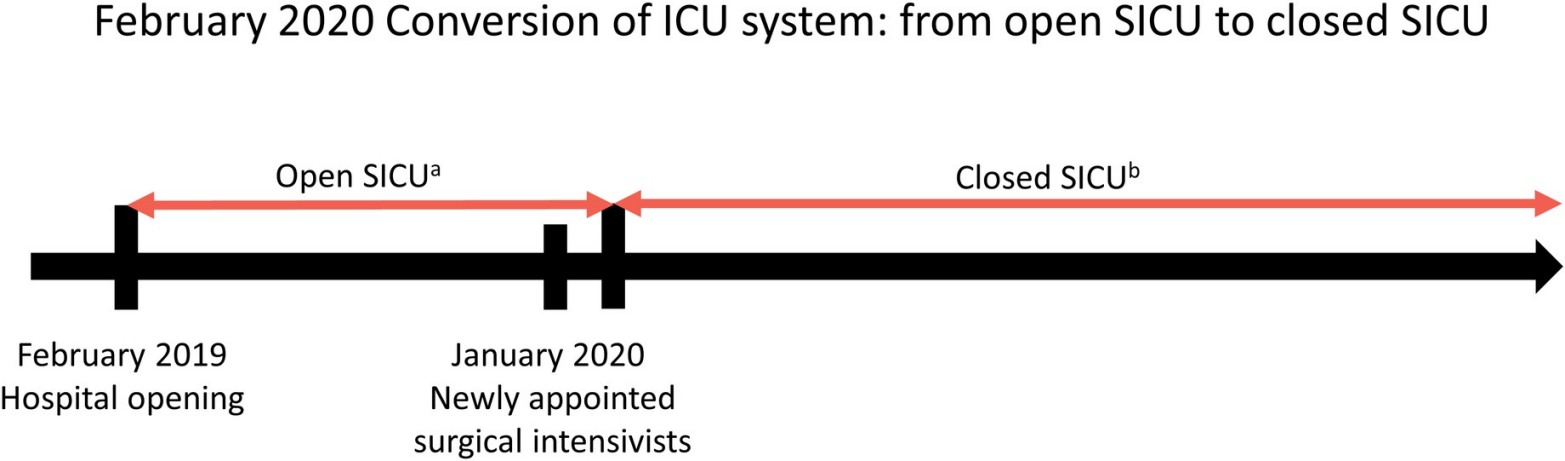
Conversion history of the ICU operating system of the institution where the study was conducted. *SICU*, surgical intensive care unit. ^a^Open SICU: treatment was provided by each specialist in each department of general surgery, orthopedic surgery, urology, obstetrics/gynecology, etc. without residents. ^b^Closed SICU: ICU treatment was provided by surgical intensivists after transferring to Critical Care Medicine department.

Our institution converted the surgical ICU system from “open” to “closed” in February 2020. We conducted this study on patients admitted to the surgical ICU from March 2019 to February 2022, and the enrolled patients were classified into the OSICU (191 patients) and CSICU (560 patients) groups at that time from March 2019 to February 2022 (Fig 1).

All enrolled patients were included in the department of surgery. Patients who agreed to discontinue “life-sustaining treatment” or to “do not resuscitate” were excluded. The OSICU group was defined by patients who received ICU management by the department that performed the surgery. The CSICU group was defined by patients who were transferred to the department of critical care medicine after surgery and managed by the intensivist during their ICU stay.

The following data were retrospectively collected on the admission day to evaluate the clinical characteristics and severity between the two groups: sex, age, body mass index (BMI), acute physiology and chronic health evaluation (APACHE) II score, sequential organ failure assessment (SOFA) score, proportion of sepsis, application of mechanical ventilator, application of CRRT, comorbidities, vital signs, and laboratory results. We also investigated the overall mortality, 28-day mortality, duration of ICU stay, duration of total hospitalization, duration of mechanical ventilator application, duration of CRRT application, net input/output (I/O) balance, and duration of norepinephrine use to evaluate the clinical outcome.

Next, we examined the total fluid input, colloid supply, transfusion, vasopressor use, analgesic use, and sedative agent use to compare the differences in ICU management between the two groups.

Finally, we examined the operating status of the ICU during the data collection period, which included the bed occupancy rate, bed turnover rate, and ICU readmission rate within 48 h.

### Statistical analysis

Categorical variables were analyzed using the chi-squared test and expressed as numbers and percentage. Continuous variables were analyzed using Student’s *t*-test and expressed as mean ± standard deviation. Factors associated with the mortality of surgical patients in the ICU were analyzed using the forward conditional method of binary logistic regression. All statistical analyses were conducted using the IBM SPSS software, version 26.0 (SPSS Inc., Chicago, IL, USA).

### Ethics approval and consent to participate

The study was approved by the Institutional Review Board (approval number: SEUMC 2020-10-024-004) and waived the informed consents due to the retrospective study.

## Results

### Patient characteristics

**Fig 2:**
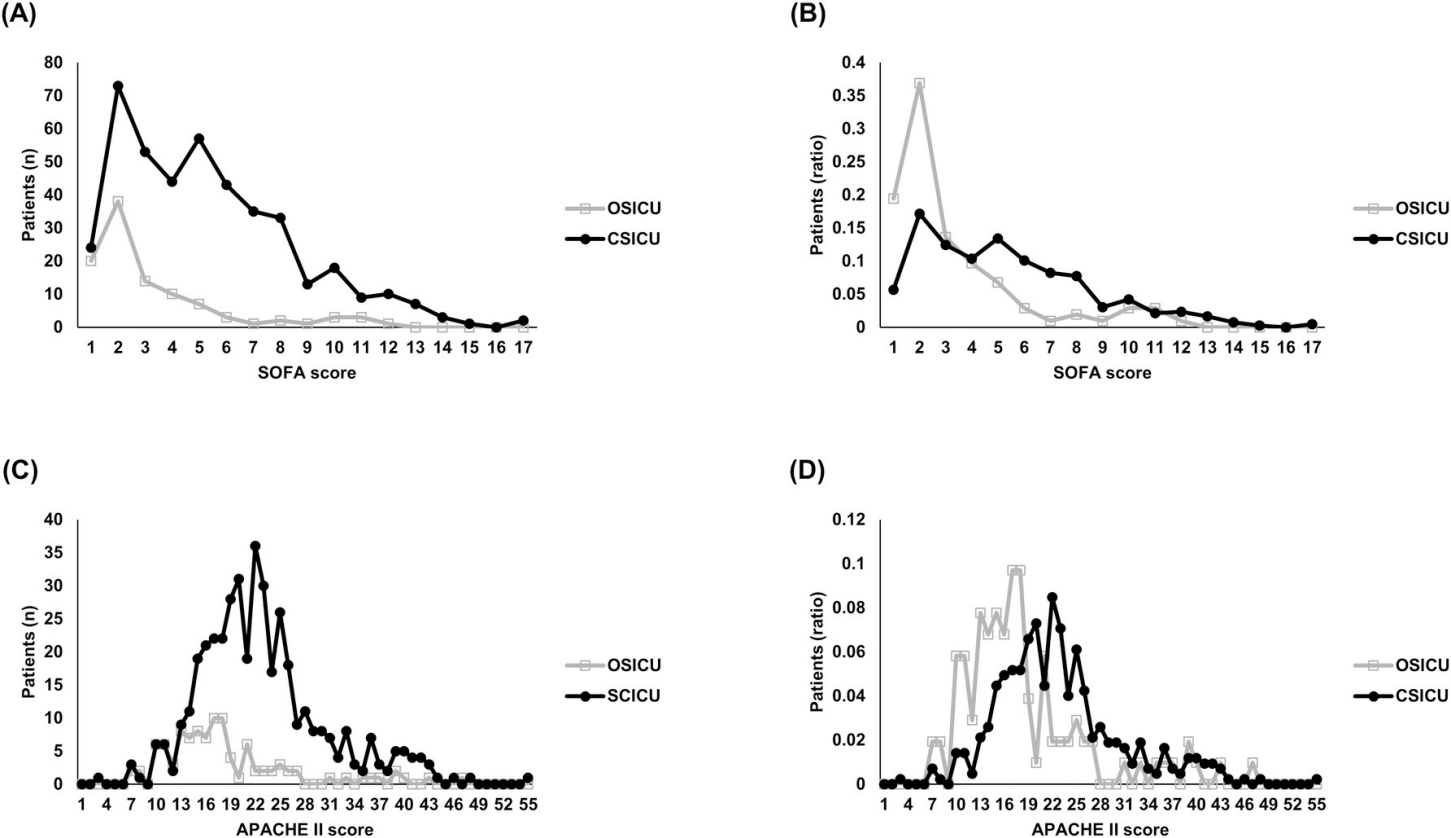
Distribution of SOFA score and APACHE II score between OSICU and CSICU groups. SOFA, sequential organ failure assessment; APACHE II, acute physiology, and chronic health evaluation II; OSICU, open surgical intensive care unit; CSICU, closed surgical intensive care unit. (A) Distribution of patients (n) by group according to SOFA score. (B) Distribution of patients (ratio) by group according to SOFA score. (C) Distribution of patients (n) by group according to APACHE II score. (D) Distribution of patients (ratio) by group according to APACHE II score.

The mean age of the patients was 67.3 ± 16.10 years in the OSICU group and 72.2 ± 15.01 years in the CSICU group, indicating a statistically significant difference. The APACHE II score was 21.8 ± 7.65 in the CSICU group, which was higher than the score 17.4 ± 7.97 in the OSICU group (*p* < 0.05). The SOFA scores were 2.0 ± 2.29 in the OSICU group and 4.1 ± 3.06 in the CSICU group (*p* < 0.05). The distribution of patients according to SOFA and APACHE II scores between the two groups is shown in Fig 2. Sepsis was found in 7.9% of patients in the OSICU group and in 12.5% of patients in the CSICU group (*p* = 0.080). Among the enrolled patients, 636 patients were admitted to the ICU immediately after surgery. Table 1 shows a comparison of the demographic and clinical characteristics between the two groups.

**Table 1:** Demographics and clinical characteristics of surgical intensive care unit patients.

Variable	OSICU group (n = 191)	CSICU group (n = 560)	*p*
Male (%)	106 (55.5)	279 (49.8)	0.175
Age (years)	67.3 ± 16.10	72.2 ± 15.01	< 0.05
BMI (kg/m^2^)	26.57 ± 34.174	23.49 ± 4.856	0.219
APACHE II score	17.4 ± 7.97	21.8 ± 7.65	< 0.05
SOFA score	2.0 ± 2.29	4.1 ± 3.06	< 0.05
Comorbidities (%)			
Hypertension	104 (54.5)	316 (56.4)	0.634
Diabetes mellitus	68 (35.6)	173 (30.9)	0.229
Heart disease	26 (13.6)	80 (14.3)	0.818
Lung disease	3 (1.6)	4 (0.7)	0.288
Liver disease	0 (0)	0 (0)	
Kidney disease	5 (2.6)	12 (2.1)	0.703
Brain disease	0 (0)	30 (5.4)	< 0.05
Malignant disease	10 (5.2)	90 (16.1)	< 0.05
Social history (%)			
Alcohol history	47 (24.6)	90 (16.1)	< 0.05
Smoking history	56 (29.3)	88 (15.7)	< 0.05
Sepsis or septic shock (%)	15 (7.9)	70 (12.5%)	0.080
Postoperative ICU admission (n = 636, %)	158 (93.5)	448 (95.9)	0.200
General Surgery (%)	123 (72.8)	326 (69.8%)	< 0.05
Orthopedic Surgery (%)	22 (13.0)	100 (21.4%)	
Urology (%)	6 (3.6)	24 (5.1)	
Obstetrics and Gynecology (%)	7 (4.1)	11 (2.4)	
Plastic Surgery (%)	10 (5.9)	6 (1.3)	
Oral and Maxillofacial Surgery (%)	1 (0.6)	0 (0)	

OSICU, open surgical intensive care unit; CSICU, closed surgical intensive care unit; BMI, body mass index; APACHE II, acute physiology, and chronic health evaluation II; SOFA, sequential organ failure assessment; CRRT, continuous renal replacement therapy; ICU, intensive care unit

### Clinical outcome

The clinical outcome results between the two groups are presented in Table 2. The overall mortality rates were 2.1% in the OSICU group and 2.0% in the CSICU group (*p* = 0.912). No significant differences were observed between the two groups in the total length of hospital stay, ICU stay, and duration of mechanical ventilation. CRRT was not applied in the OSICU group, but in the CSICU group, CRRT was applied for a mean of 4.0 ± 3.57 days. The duration of norepinephrine use for blood pressure maintenance was 8.6 ± 21.90 days in the CSICU group and 0.4 ± 2.32 days in the OSICU group (*p < 0*.*05*).

**Table 2:** Clinical outcomes of open and closed surgical intensive care unit.

Variable	OSICU group (n = 191)	CSICU group (n = 560)	*p*
Overall mortality (%)	4 (2.1)	11 (2.0)	0.912
28-day mortality (%)	2 (1.0)	10 (1.8)	0.482
Hospitalization period (days)	22.8 ± 22.99	23.6 ± 22.81	0.695
Duration of ICU stay (days)	4.7 ± 8.72	6.3 ± 14.77	0.146
Mechanical ventilator (%)	24 (12.6)	103 (18.4)	0.064
Period of mechanical ventilator application (days)	7.7 ± 11.42	10.0 ± 21.79	0.636
CRRT (%)	0 (0)	19 (3.4)	< 0.05
Periods of CRRT application (days)	None	4.0 ± 3.57	-
Use of norepinephrine (%)	11 (5.8)	233 (41.6)	< 0.05
Duration of norepinephrine (days)	0.4 ± 2.32	8.6 ± 21.90	< 0.05

OSICU, open surgical intensive care unit; CSICU, closed surgical intensive care unit; ICU, intensive care unit; CRRT, continuous renal replacement therapy; I/O, intake/output

### Analysis of factors affecting mortality

The factors influencing mortality in the target patients were investigated using a logistic regression analysis (Table 3), which yielded an odds ratio of 0.089 (95% CI: 0.014–0.568; *p* < 0.05) in the CSICU group. Moreover, the odds ratios were 22.834 (95% CI: 1.662–313.663; *p* < 0.05) for patients with kidney disease, 24.912 (95% CI: 3.063–202.611; *p* < 0.05) for patients on ventilation, and 44.940 (95% CI: 5.989–307.870; *p* < 0.05) for patients receiving CRRT.

**Table 3:** Binary logistic regression analysis of factors associated with mortality.

Variable	B	Exp (B)	95% CI	*p*
Group^a^	-2.419	0.089	0.014–0.568	< 0.05
Comorbidity (kidney disease)	3.128	22.834	1.662–313.663	< 0.05
Mechanical ventilator	3.215	24.912	3.063–202.611	< 0.05
CRRT	3.760	44.940	5.989–307.870	< 0.05

OSICU, open surgical intensive care unit; CSICU, closed surgical intensive care unit; CRRT, continuous renal replacement therapy

Adjusted for age, APACHE II score, SOFA score, comorbidities, norepinephrine use, sepsis, total input of day 1 and total input of day 2.

^a^Group: OSICU group/CSICU group

### Comparison of ICU care between CSICU and OSICU groups

The amount of fluid and albumin supplied on the 1st day of admission to the ICU was significantly higher in the CSICU group. There was no statistical difference in the total fluid input on day 2 of ICU admission between the two groups (*p* = 0.271), but the intake of albumin was significantly higher in the CSICU group. Norepinephrine was used in 233 patients (41.6%) in the CSICU group and in 11 patients (5.8%) in the OSICU group (*p* < 0.05). Vasopressin was used in 44 patients (7.9%) to increase blood pressure in the CSICU group, but not in the OSICU group. Epinephrine was also used to increase blood pressure in 18 patients (3.2%) in the CSICU group, but not in the OSICU group. Arterial cannulation for measuring arterial blood pressure was applied in 501 patients (89.5%) in the CSICU group and in 128 patients (67.0%) in the OSICU group (*p* < 0.05). The usage pattern of opioids and sedative agents was also different between the two groups. Fentanyl and remifentanil were more used in the CSICU group for analgesic effect (*p* < 0.05). For sedation, dexmedetomidine was also widely used in the CSICU group (*p* < 0.05). A detailed comparison of ICU care between the two groups is shown in Table 4.

**Table 4:** Comparison of treatment strategy between open and closed surgical intensive care unit.

Variable	OSICU group (n = 191)	CSICU group (n = 560)	*p*
Fluid resuscitation			
1st day of ICU admission			
Total Intake (ml)	3439.5 ± 1889.50	3836.1 ± 2030.54	< 0.05
Net I/O on 1st day of ICU admission (ml/kg/day)	27.9 ± 25.40	33.7 ± 38.45	< 0.05
20% albumin 100 ml (ml)	29.3 ± 56.93	40.9 ± 67.59	< 0.05
2nd day of ICU admission			
Total Intake (ml)	2752.0 ± 988.31	2854.4 ± 1145.75	0.271
Net I/O the 2nd day of ICU admission (ml/kg/day)	12.3 ± 19.38	11.4 ± 23.45	0.629
20% albumin 100 ml (ml)	38.7 ± 62.97	49.3 ± 60.11	< 0.05
Vasopressors			
Use of norepinephrine (%)	11 (5.8)	233 (41.6)	< 0.05
Maximum norepinephrine infusion rate (μg/kg/min)	0.13 ± 0.146	0.08 ± 0.107	0.112
Use of vasopressin (%)	None	44 (7.9)	< 0.05
Maximum vasopressin infusion rate (IU/min)	None	0.03 ± 0.046	
Use of epinephrine (%)	None	18 (3.2)	< 0.05
Maximum epinephrine infusion rate (μg/kg/min)	None	0.13 ± 0.099	
Analgesics and sedatives (%)			
Fentanyl	101 (52.9)	482 (86.1)	< 0.05
Remifentanil	36 (18.8)	151 (27.0)	< 0.05
Propofol	7 (3.7)	34 (6.1)	0.206
Dexmedetomidine	2 (1.0)	401 (71.8)	< 0.05
Catheterization			
Central catheter (%)	4 (2.1)	20 (3.6)	0.316
Location of central catheter (%)			
Subclavian vein	2 (50.0)	7 (35.0)	
Internal jugular vein	2 (50.0)	9 (45.0)	0.601
Femoral vein	None	4 (20.0)	
Arterial cannulation (%)	128 (67.0)	501 (89.5)	< 0.05
Transfusion			
1st day of ICU admission			
Red blood cell (unit)	0.7 ± 2.68	1.1 ± 2.49	0.060
Fresh frozen plasma (unit)	0.2 ± 1.53	0.4 ± 1.44	0.298
Platelet concentration (unit)	0.3 ± 2.45	0.1 ± 0.77	0.362
2nd day of ICU admission			
Red blood cell (unit)	0.2 ± 0.60	0.3 ± 0.84	0.134
Fresh frozen plasma (unit)	0.1 ± 0.57	0.0 ± 0.35	0.241
Platelet concentration (unit)	0.1 ± 0.85	0.1 ± 0.54	0.426
Others			
Antacid (%)	133 (69.6)	543 (97.0)	< 0.05
Low-molecular-weight heparin for DVT prophylaxis (%)	40 (20.9)	107 (19.1)	0.581
Intermittent pneumatic compression for DVT prophylaxis (%)	1 (0.5)	518 (92.5)	< 0.05
Benzydamine hydrochloride or chlorhexidine gluconate solution for oral care (%)	10 (5.2)	539 (96.3)	< 0.05
High-flow nasal cannula (%)	None	212 (37.9)	< 0.05

OSICU, open surgical intensive care unit; CSICU, closed surgical intensive care unit; ICU, intensive care unit; DVT, deep vein thrombosis

### Changes in ICU operation before and after the application of the CSICU system

Table 5 shows the changes in ICU operation after the application of the CSICU system. The bed occupancy rate of ICU was 97.46% during the application period of the CSICU system, which was higher than the rate of 86.2% during the application period of the OSICU system. The bed turnover rate was 85.48% during the application period of the CSICU system, which was higher than the rate of 78.87% during the application period of the OSICU system. The ICU readmission rate within 48 h was 2.0% during the OSICU system application period, which decreased to 1.43% during the CSICU system application period.

**Table 5:** Comparison of ICU operating efficiency according to ICU operating system.

Variable	OSICU period	CSICU period
Bed occupancy rate (%)	86.20	97.46
Bed turnover rate (%)	78.87	85.48
Readmission rate within 48 h (%)	2.00	1.43

OSICU, open surgical intensive care unit; CSICU, closed surgical intensive care unit

## Discussion

To our knowledge, our study is the first in Korea to explore the effects of the CSICU system. We found that the CSICU system led by intensivists exerts a positive impact on patients’ prognosis and ICU operation [10–15]. This study chronologically investigated the effect of the application of the CSICU system on the clinical outcome of only surgical patients to confirm its usefulness in the surgical ICU.

In February 2020, since our institution had changed the surgical ICU from an open system to a closed system, there has been a large change in ICU operation. In particular, bed occupancy and bed turnover rates increased (bed occupancy rate: OSICU:CSICU = 86.20%:97.46%; bed turnover rate: OSICU:CSICU = 78.87%:85.48%; Table 5), for which there are some reasons. First, the ICU beds were operated efficiently as the admission and discharge of ICU patients were completely controlled by the intensivist. This not only promotes the public interest of efficiently managing limited medical resources but also has the effect of reducing unnecessary medical expenses. It also reduces long-term hospitalization and induces rapid recovery of patients. Second, it is believed that surgeons made more active decisions regarding hospitalization and surgery for critically ill patients as the burden of managing them was reduced after the change in the ICU system. This can be inferred from the fact that scores reflecting the severity of patients, such as the APACHE II and SOFA scores, also increased significantly in the CSICU group after the change of the ICU system (Table 1 and Fig 2). The change in the ICU system provided an environment capable of treating several patients with high severity.

Not only the efficiency of ICU bed operation but also the clinical outcomes were improved. No significant difference was observed in the mortality rate between the OSICU and CSICU groups, but the scores indicating severity such as the APACHE II and SOFA scores showed significant differences between the two groups (Table 1 and Fig 2). Therefore, to investigate the effect of the ICU system on mortality, we performed a binary logistic regression with conditional (forward) method adjusting for age, comorbidities, norepinephrine use, and sepsis, including the abovementioned scores, which generally affect clinical outcomes. Consequently, as observed in previous studies related to the CSICU system, its application resulted in a mortality odds ratio of 0.089 (95% CI: 0.014–0.568; *p* < 0.05), which indicated a more favorable result in the CSICU group (Table 3).

We also analyzed the treatment factors that caused these differences in the clinical outcome, and in fact, the two groups showed several differences in treatment (Table 4). First, vasopressor use for blood pressure maintenance was different between the two groups. A distinctive difference was that there was no use of vasopressin in the OSICU group. The difference in vasopressor use between the two groups is considered to be due to the treatment performed in the OSICU system by surgeons not exposed to or unfamiliar with the latest critical care and surviving sepsis campaign guidelines. In the 2021 surviving sepsis campaign, the use of norepinephrine as the first-line drug for blood pressure elevation was recommended, which has already been agreed upon in critical care medicine. Furthermore, the use of vasopressin as a combination therapy is recommended. Numerous studies have already reported the effect of the concomitant administration of vasopressin for blood pressure maintenance [20–22]. Research is still ongoing on the optimal protocol for the use of vasopressors to maintain blood pressure in patients with septic shock [23]. The fact that there was no use of vasopressin with consensus in the OSICU group indicates the need to consider the application of an optimal ICU system.

Colloid supply was significantly higher in the CSICU group on both the 1st and 2nd day of admission. Herrmann et al. reported that a low serum albumin level measured within 48 h of admission leads to an increase in hospital stay and mortality [24]. Among the patients enrolled in this study, sepsis was diagnosed in 7.9% in the OSICU group and 12.5% in the CSICU group, but the respective mean SOFA scores were 2.0 ± 2.29 and 4.1 ± 3.06, and both groups showed organ failure scores that met the sepsis-3 diagnostic criteria. The surviving sepsis campaign announced in 2021 strongly recommends the use of crystalloid rather than colloid for sepsis and early resuscitation of septic shock; however, this does not imply that the use of colloid itself is harmful. Because vascular permeability increases in sepsis, large molecules such as albumin can leak into the interstitium [25]. Several reports have indicated that correcting low serum albumin levels improves clinical outcomes [26–31]. However, although colloid use in critically ill patients, especially sepsis, still needs more research, it can be stated that the more active colloid supply in the CSICU group is a specific difference.

The two groups also showed significant differences in the method of hemodynamic monitoring. Representatively, arterial cannulation, which can accurately monitor blood pressure in real time, was performed in 89.5% of patients in the CSICU group and in 67.0% of patients in the OSICU group (Table 4). In our institution where the study was conducted, arterial cannulation is performed on all patients during the anesthesia stage before surgery. However, there are cases where arterial cannulation cannot be maintained and removed in the ICU after surgery due to various reasons. Blood pressure measurement using a noninvasive cuff is inaccurate in critically ill patients with high severity, and it becomes more pronounced in critically ill patients with severity such as shock [32–34]. If vasopressors need be used due to low blood pressure, arterial cannulation should be performed in real time for hemodynamic monitoring, and a mean arterial blood pressure of ≥65 mmHg recommended in the 2021 surviving sepsis campaign should be maintained [35]. The application of the CSICU system can improve clinical outcomes by adopting an accurate method based on evidence in terms of patient monitoring as well as treatment of critically ill patients.

The use of low-molecular-weight heparin (LMWH) for the prevention of deep vein thrombosis (DVT) was not different between the two groups, but the use of intermittent pneumatic compression (IPC) revealed a significant difference. Critically ill patients have a high risk for DVT and pulmonary thromboembolism (PE) [36–38]. Because PE can lead to fatal consequences, prevention of DVT should always be considered in critical care management. Despite reports that the use of LMWH for postoperative DVT prevention does not increase the risk of bleeding [39], several surgeons are hesitant to use it. After the application of the CSICU system, there were positive results in the surgical ICU through the establishment of a protocol for the use of IPC for all postoperative critical patients in the absence of special contraindication.

Our study is valuable as it investigated the effects of differences in ICU operating systems targeting surgery departments. With the emergence of the positive effects of the CSICU system, it is essential to transform the ICU operating system throughout medical care in Korea. In particular, in countries such as Korea that have a problem of the lack of medical staff in the surgery department [6, 19], it is believed that the activation of surgical intensivists and the application of CSICU systems can be a solution. Additional studies must be conducted on the positive effects of a dedicated ICU consisting of an intensivist and a CSICU system, and medical policies must be promoted based on this need. In this study, we investigated the difference in clinical outcomes and management methods for critically ill patients between the open system and closed system of the surgical ICU. However, our study has some limitations. First, there was a significant difference in severity between the two groups. However, this bias was statistically corrected and analyzed, and in particular, the significantly better clinical outcome was confirmed in the CSICU group, wherein the patients had a much higher severity, because of the superiority of the CSICU system. Second, this study was conducted in a single institution. However, it is also considered meaningful that favorable results were obtained when only one ICU system was changed in the same environment, such as the same medical staff, paramedical staff, and the same medical equipment in a single institution. In the future, based on our research, we will analyze the current status of surgical ICUs in multiple institutions in Korea and prepare a basis for the advantages of applying a closed system to surgical ICUs.

## Conclusion

A positive clinical outcome was confirmed after applying a closed system to the surgical ICU. Currently, Korea is experiencing a very serious shortage of surgeons. In 2022, the resident application rate for surgery department in Korea was only 60%. The application of the closed system to the surgical ICU will enable providing more specialized critical care despite the limited surgical resources, and it is expected that this will result in improved clinical outcomes for critically ill surgical patients. In addition, this study will surely help countries with similar medical systems to Korea consider ICU operating systems to improve the quality of critical care.
